# A Comparison of Laparoscopic Surgery and Open Surgery in Liver Resections: A Single-Center Experience

**DOI:** 10.5152/eurasianjmed.2023.23119

**Published:** 2023-10-01

**Authors:** Salih Kara, Ercan Korkut, Nurhak Aksungur, Necip Altundaş, Gürkan Öztürk, Enes Ağırman, Metin Yıldız

**Affiliations:** 1 Department of Transplantation Center, Atatürk University Faculty of Medicine, Erzurum, Turkey; 2 Department of General Surgery, Erzurum City Hospital, Erzurum, Turkey; 3 Department of General Surgery, Atatürk University School of Medicine, Erzurum, Turkey

**Keywords:** Hepatectomy, laparoscopic liver resection, liver neoplasms

## Abstract

**Objective:**

With the developments in patient management and the increase in surgical experience, the use of laparoscopy in liver resections has become widespread. However, with the consensus meetings and international recommendations, laparoscopic liver resections have been tried to be standardized. We aimed to present this laparoscopic liver resection experience by comparing open and laparoscopic techniques.

**Materials and Methods:**

Patients who underwent liver resections between 2015 and 2022 were retrospectively screened and divided into 2 groups as laparoscopic liver resections and patients who underwent liver resection with open surgery. Indications, resection techniques, operative times, length of hospital stay, early hospital mortality, and complications were compared between both groups using statistical methods.

**Results:**

Laparoscopic surgery was performed in 31 (14%) patients, and open surgery was performed in 189 (86%). The mean operation time was 316 ± 168.2 minutes in patients who underwent laparoscopic liver resection. It was 329.4 ± 123.6 in the open surgery group. The length of hospital stay was 11.6 ± 4.9 days in patients who underwent laparoscopic liver resection, while it was 19.7 ± 12.1 days in patients who underwent open surgery. The difference between the length of hospital stay was statistically significant (Mann–Whitney *U*-test, *P* = .00). There was no difference between the 2 groups in terms of complications and early mortality.

**Conclusion:**

Laparoscopic liver resections are a safe method that can be applied in 3 or less segment resections. As the experience of the surgical team increases, it can be safely applied for major hepatectomies.

**Main Points**

- Laparoscopic liver resections can be safely performed in patients who will undergo 3 or less segment resections.
- Laparoscopic major hepatectomies should be performed by experienced surgeons in experienced centers.
- Laparoscopic liver resections provide earlier recovery by reducing the immobilization and pain of the patients.

## Introduction

Although minimally invasive liver resections were first performed in the 1990s, the development of laparoscopic surgery of the liver occurred later.^1^ Difficulties in techniques in liver surgeries and intraoperative patient management caused a delay in the development of laparoscopy. With the advancements in patient management over time, its use has become widespread in recent years. Correspondingly, the role of laparoscopic liver resection (LLR) has begun to be established for liver malignancies, particularly hepatocellular carcinoma (HCC), which has been confirmed by consensus meetings and international recommendations.^2,3^ A meta-analysis between laparoscopic and open liver resection for HCC showed shorter length of hospital stay and less intraoperative blood loss in LLR. Similar results were found between them in terms of patient survival and disease-free survival.^4,5^ However, laparoscopic anatomic liver resection techniques have not been fully standardized. Notwithstanding, while laparoscopic techniques continue to develop, agents such as indocyanine green (ICG) have been introduced in terms of lesion imaging.^6^ Indocyanine green fluorescence imaging adds a lot of precision to laparoscopic anatomical hepatectomy. The success of segmental staining is useful for interpreting the resection margin. However, the technique needs to be standardized and validated by further studies.^6,7^

In our clinic, open surgery of hepatobiliary cases has been standardized in the last 20 years. In addition to open surgery, laparoscopic liver resections have been performed with high quality in the recent years. Therefore, we wanted to present our experience and contribute to the literature by comparing open and laparoscopic techniques of liver resection.

## Materials and Methods

This study was approved by the Atatürk University Medicine Ethics Committee (Date: December 1, 2022; Approval No.: 10-77). All procedures in this study involving human participants were performed in accordance with the 1964 Declaration of Helsinki and its later amendments. Informed consent form was obtained preoperatively from all patients who underwent surgery and included in the study.

Patients who underwent liver resections in the Department of General Surgery Hepatobiliary Service at Atatürk University between 2015 and 2022 were retrospectively screened and included in the study. Patients who underwent donor hepatectomy for a liver transplant with a living donor were excluded from the study. The patients who underwent donor hepatectomy were completely healthy individuals, the liver parenchyma reserves are better and of higher quality. So it were not included in the study because it may affect the results of the study. Accordingly, a total of 393 patients were reviewed. Due to donor hepatectomy, 166 participants were excluded from the study. Seven autotransplantation cases, which we thought may affect the outcome of the study due to the surgical technique and intraoperative procedures, were excluded from the study. As a result, 220 patients were included in the study and examined.

Patients included in the study were divided into 2 groups: laparoscopic liver resections and patients who underwent liver resection with open surgery. Demographic data (age, gender), indications, resection techniques, duration of surgery, length of hospital stay, early hospital mortality, and complications were recorded for each group. Operations that included surgical resection techniques were right hepatectomy, left hepatectomy, extended right hepatectomy, extended left hepatectomy, and segmentectomy (resections performed below 3 segments). Additionally, the mass localizations of the patients with a mass in the liver parenchyma were recorded. Patients who underwent resection for perihilar cholangiocarcinoma were categorized according to the bismuth classification and recorded.^8^

### Patient Management

Patients generally applied to our center from another hospital. Therefore, they had radiological imaging on their first visit. Additional imaging was performed according to the preliminary diagnosis of the patients who applied to our center. Triphasic abdominal computed tomography was performed in all patients to evaluate the vascular structures and to make volumetric measurements. Dynamic magnetic resonance imaging images were obtained by administering hepatospecific agent according to clinical necessity. Patients whose laboratory and radiological examinations were completed were evaluated in our hepatobiliary council, which also attended by medical oncology, radiology, pathology and gastroenterology experts, and the management of the patients and surgical technique planning were made. The operated patients were followed up in the intensive care unit on the first postoperative day and were taken to the service according to their clinic.

### Surgical Procedure

All patients were evaluated according to the Eastern Cooperative Oncology Group performance score, and resection was planned for patients with masses with a score of 0-2, where technically a negative surgical margin could be obtained within oncological principles. Due to the risk of postoperative liver failure during the preoperative period, appropriate planning was also performed by measuring the remnant liver volume with volumetric measurements when necessary. Accordingly, the associating liver partition and portal vein ligation for staged hepatectomy technique, a 2-stage surgical resection procedure, was performed in some cases. In the remaining cases, right and left hepatectomy, extended right and left hepatectomy, left lateral sectionectomy, and parenchyma-sparing resections were performed according to the location of the lesion. In addition to resection, biliary tract reconstruction procedures, which may increase morbidity, were also performed on patients who underwent extended hepatectomy.

Median incision or Makuuchi incision was used according to the localization of the lesion in the liver. Especially in malignant patients, resection was started after exploration in terms of intra-abdominal tumor spread. After securing the artery, portal vein, and hepatic vein of the remaining liver, parenchymal transection was performed. If the size of the mass is small and the parenchyma to be resected is small, resection was completed only with bipolar cautery and surgical energy devices. In addition to these devices, Cavitron Ultrasonic Surgical Aspirator was used in major hepatectomies. During parenchymal transection, portal vein branches and minor bile ducts were closed with clips or primary sutured according to their size. Right–left portal vein branches and hepatic artery branches were closed with vascular clamps and primary sutured, while hepatic vein branches were closed using a vascular stapler.

### Statistical Analysis

Quantitative parameters were calculated as arithmetic mean ± SD and as numbers and percentages for categorical variables. The distribution of numerical data was evaluated with the Shapiro–Wilk test, the Kolmogorov–Smirnov test, and histogram graphics. The Mann–Whitney *U*-test, which is a nonparametric test, was utilized to analyze the relationship between categorical variables and postoperative results as the numerical data were not normally distributed. The chi-square test was used to compare categorical data. The data were analyzed at a 95% confidence interval and the p-significance value was accepted as <.05. Statistical Package for the Social Sciences Statistics, version 20.0, software was (IBM SPSS Corp.; Armonk, NY, USA) used for statistical analysis.

## Results

A total of 220 patients were examined in our study. Of these patients, 31 (14%) underwent laparoscopic surgery, and 189 (86%) underwent open surgery. The patients consisted of 122 (55.5%) female and 98 (44.5%) male patients. Of the patients who underwent laparoscopic surgery, 19 (61.3%) were female and 12 (38.7%) were male. Among the patients who underwent open surgery, 103 (54.5%) were female and 86 (45.5%) were male. The mean age of the patients in the laparoscopic surgery group was 45.5 ± 12.3, while the mean age of the patients in the open surgery group was 53 ± 16.7. The patients in both groups mostly underwent surgery with the diagnosis of alveolar echinococcosis. While 14 (45.2%) of the patients in the laparoscopic group had alveolar echinococcosis, 67 (35.4%) of the patients in the open surgery group had alveolar echinococcosis. Five (16.1%) patients underwent laparoscopic resection for a metastatic liver tumor. Open surgery was performed in 47 (24.9%) patients due to metastatic tumors. In total, 35 (67.3%) of the metastatic tumors were metastases of colorectal cancer. In the laparoscopic group, 21 (67.7%) patients underwent segmentectomy as a surgical procedure, while 55 (29.1%) patients in the open surgery group underwent segmentectomy. Furthermore, laparoscopic right hepatectomy was performed in 2 (6.5%) patients, while laparoscopic left hepatectomy was performed in 3 (9.7%) patients in the laparoscopic surgery group. Complications developed in 34 (18%) patients in the open surgery group, while complications developed in 2 (6.5%) patients in the laparoscopic group (Table 1). The complication rate for all liver resections was 16.3%. In-hospital death in the early period was observed in 13 (5.9%) patients. All of the patients who died underwent liver resection with the open technique. While 4 of the patients who died were due to complications of small for size syndrome, the remaining 9 were due to sepsis.

In our study, no statistically significant difference was found in the operation time in comparisons between the 2 groups (Mann–Whitney *U*-test; *P* = .49). The mean operation time was 316 ± 168.2 minutes in patients who underwent laparoscopic liver resection. It was 329.4 ± 123.6 in the open surgery group. The difference between the 2 groups in terms of duration of hospital stay was statistically significant (Mann–Whitney *U*-test; *P* = .00). The length of hospital stay was 11.6 ± 4.9 days in patients who underwent laparoscopic liver resection, while it was 19.7 ± 12.1 days in patients who underwent open surgery. Accordingly, patients who underwent laparoscopic surgery had a shorter hospital stay. There was no statistically significant difference between the 2 groups in primary diagnosis, lesion location, or gender (Fisher’s exact test; *P* = .19, .59, and .3, respectively). Although complications and early mortality were more common in open surgery, this difference was not statistically significant (*P* = .17 and *P* = .13, respectively; Table 1). There was a significant difference in surgical technique between the 2 groups (*P* = .03; Table 1). This result shows that open surgeries were preferred more for major resections.

## Discussion

Due to the complexity and technical limitations of liver surgery, its development in the field of minimally invasive surgery has continued for many years. In recent years, liver surgery has been better integrated into therapeutic strategies. The use of minimally invasive surgical techniques has become more common after studies reported their advantageous aspects in terms of complications developing in the postoperative and intraoperative periods.^9^ However, conditions requiring liver resection are usually diseases that necessitate complex and multidisciplinary approaches. Surgical resections of these diseases are equally challenging. Therefore, various guidelines have been published, and it has been mutually agreed upon that a learning curve is required for the application of minimally invasive methods in liver surgeries and that it should be performed in experienced centers within certain criteria.^3^ Laparoscopic application of resections is very difficult, especially in diseases such as extrahepatic cholangiocarcinomas. In this study, we found that LLR is applicable for minor resections such as segmentectomy or lesions smaller than 5 cm. We found that it can be safely applied in hepatic alveolar echinococcosis and HCC resections. In addition, we found that there is a faster recovery process in the postoperative period in minimally invasive liver surgery.

The best indications for LLR were determined to be lesions measuring 5 cm or less. In addition, laparoscopic surgery for left lateral sectionectomy was accepted as a standard technique.^3,10^ Left lateral and right anterior segment lesions seem to be technically easier now. It is reported in Southampton guidelines that experienced surgeons can perform laparoscopic resections of lesions in the posterosuperior segments as well as anterolateral segment resections.^3^ Likewise, resections that are technically challenging, such as recurrent lesions, major hepatectomies, and 2-stage resections, should be performed by experienced surgeons. Laparoscopic liver resection is especially recommended for patients in the low- or medium-challenge class. However, it has no advantage over open surgery due to the longer operation time for.^11,12^ Laparoscopic liver resection has also been performed for larger lesions in recent years, although lesions smaller than 5 cm are recommended for LLR. A study on LLR for colorectal liver metastases showed that LLR is safe and effective for tumors greater than 10 cm in diameter. Similarly, LLR performed on benign lesions larger than 10 cm has also been reported.^13,14^ In our study, most of the cases that underwent LLR were segmentectomy and left lateral sectionectomy. Considering the performance rates, we concluded that segmentectomies are mostly preferred for laparoscopic surgeries. Our results were aligned with the guidelines in terms of surgical resection technique, like similar studies in the literature.^15^ In our study, it was observed that open surgery was generally preferred in 2-stage hepatectomies and major hepatectomies. Since the rate of major surgery is higher in the open surgery group and minor resections are preferred more in laparoscopic surgery, we can say that the LLR procedure is performed in our center in accordance with the treatment guidelines in terms of surgical technique. The statistical significance of these differences confirms our opinion (Table 1, Pearson’s correlation and chi square; *P* = .03).

The main criteria for the application of laparoscopic liver resection include the size, location and proximity of the tumor to vascular structures rather than the primary indication. Furthermore, liver reserves are also determining factors, particularly in patients with cirrhosis. In fact, this is a decisive factor not only for LLR but also for open surgery. Resection is the first-line treatment for hepatocellular carcinoma (HCC) stage 0-A according to the Barcelona Clinic Liver Cancer (BCLC) grading system.^16,17^ Therefore, it is necessary to determine the remnant liver reserve in the preoperative period. In addition, both robotic and laparoscopic hepatectomies have been found to be safe and effective in patients with BCLC grade 0-A HCC when compared to open hepatectomy.^11^ By paying attention to these criteria, liver resections were performed in the treatment of hepatocellular carcinoma, gallbladder carcinoma, and intrahepatic or bismuth type 3 perihilar cholangiocarcinoma, among the malignancies detected in the preoperative period in our center. In addition, liver resection procedures were applied in the treatment of hepatic alveolar echinococcosis, which is endemic in our region. Using scientific guidelines, we also performed resection of metastatic liver masses according to the management of primary tumors. In our series, liver resection was performed the most in total for hepatic alveolar echinococcal disease. Likewise, LLR was mostly performed on patients in this group. Liver resections performed for metastatic liver disease and hepatocellular carcinoma were also common indications. As our center is located in an endemic area for alveolar echinococcal disease, the majority of resections we perform are for hepatic alveolar echinococcal disease. According to this study, 1 of the 5 patients who underwent liver resection for hepatic alveolar echinococcosis was completed laparoscopically, and no complications were observed. Accordingly, we think that LLR can be safely performed for hepatic alveolar echinococcal disease. In similar studies, PNM has been reported to be a reliable method for stage 1 hepatic alveolar echinococcosis.^6^

Laparoscopic liver resection is advantageous in several ways because it is minimally invasive and accelerates recovery. There are also meta-analysis studies that support this data. These meta-analyses concluded that the results of blood loss, perioperative blood transfusion, postoperative morbidity and mortality, and the duration of hospital stay were better in patients who underwent LLR. No statistical relationship was found between those results and the duration of the operation.^18,19^ In our study, there was no statistically significant difference in the duration of surgery and complications between open surgery and LLR, while the length of hospital stay was significantly shorter in patients who underwent LLR. We can say that minimally invasive surgeries are advantageous as they both reduce the cost and, especially, the risk of hospital-acquired infections by reducing the length of stay in the hospital.

The small number of LLRs performed in major hepatectomies and 2-stage hepatectomies and the retrospective design is the limitations of the study. However, considering the indications of the patients who underwent LLR, these limitations do not affect the reliability of the study, as the indications are similar to the guidelines and application indications in other studies in the literature. This study will contribute to future studies on this subject as the surgical experience increases and the application indications expand.

## Conclusion

Laparoscopic liver resections are a safe method that can be performed in resections of 3 or fewer segments. Laparoscopic liver resection is a surgical operation that provides a more comfortable and faster recovery. As the experience of the surgical team increases, it can be safely applied for major hepatectomies.

## Figures and Tables

**Table 1.** Patient Characteristics and Statistical Analysis

		Laparoscopic Surgery (n = 31)	Open Surgery (n = 189)	*P*
Age		45.5 ± 12.3 (25-72)	53 ± 16.7 (19-90)	**.01**
Gender	Female	19 (61.3%)	103 (54.5%)	.30
	Male	12 (38.7%)	86 (45.5%)	
Primary diagnosis	Metastatic liver tumor	5 (16.1%)	47 (24.9%)	.19^*^
	HCC	5 (16.1%)	16 (8.5%)	
	Alveolar echinococcosis	14 (45.2%)	67 (35.4%)	
	Gallbladder cancer	0	10 (5.3%)	
	Intrahepatic cholangiocarcinoma	0	4 (2.1%)	
	Perihilar cholangiocarcinoma	0	15 (7.9%)	
	Cystic echinococcosis	2 (6.5%)	11 (5.8%)	
	Focal nodular hyperplasia	2 (6.5%)	4 (2.1%)	
	Hemangioma	2 (6.5%)	7 (3.7%)	
	Hepatic adenoma	1 (3.2%)	0	
	Hepatolithiasis	0	3 (1.6%)	
	Abscess	0	3 (1.6%)	
	Liver laceration	0	2 (1.1%)	
Lesion location	Right lobe	19 (61.3%)	95 (50.3%)	.59^*^
	Left lobe	9 (29%)	45 (23.8%)	
	Bilobar	1 (3.2%)	21 (11.1%)	
	Central lobe	2 (6.5%)	11 (5.8%)	
	Caudate lobe	0	1 (0.5%)	
	Type 3A Klatskin tumor	0	8 (4.2%)	
	Type 3B Klatskin tumor	0	8 (4.2%)	
Surgical technique	Right hepatectomy	2 (6.5%)	33 (17.5%)	.03^*^
	Left hepatectomy	3 (9.7%)	22 (11.6%)	
	Extended right hepatectomy	0	12 (6.3%)	
	Extended left hepatectomy	0	5 (2.6%)	
	Segmentectomy (<3 Couinaud segment)	21 (67.7%)	55 (29.1%)	
	Left lateral sectionectomy	4 (12.9%)	19 (10.1%)	
	Extended right hepatectomy + ALPPS	0	6 (3.2%)	
	Extended left hepatectomy + ALPPS	0	1 (0.5%)	
	Parenchyma-preserving liver resection	1 (3.2%)	31 (16.4%)	
	Pericystectomy	0	4 (2.1%)	
	Caudate lobe resection	0	1 (0.5%)	
Operation time (minutes)		316 ± 168.2 (40-750)	329.4 ± 123.6 (50-660)	.49
Hospital duration (days)		11.6 ± 4.9 (4-25)	19.7 ± 12.1 (3-69)	**.00**
Complication	None	29 (93.5%)	155 (82%)	.17^*^
	CD1	2 (6.5%)	5 (2.6 %)	
	CD2	0	3 (1.6 %)	
	CD3	0	13 (6.9%)	
	CD4	0	0	
	CD5	0	13 (6.9%)	
In a hospital mortality		0	13 (6.9%)	.13^*^

ALPPS, associating liver partition and portal vein ligation for staged hepatectomy; CD1, any deviation from the normal postoperative course without the need for pharmacological treatment or surgical, endoscopic, and radiological interventions; CD2, requiring pharmacological treatment with drugs other than such allowed for grade I complications; CD3, requiring surgical, endoscopic, or radiological intervention; CD4, life-threatening complications (including central nervous system complications) requiring intermediate care/intensive care unit management; CD5, death of a patient; HCC, hepatocellular carcinoma.

^*^ *P*-value was calculated using Fisher’s exact test.
